# Editorial: Nanotechnology for Antimicrobials, Volume 2

**DOI:** 10.3389/fmicb.2022.860908

**Published:** 2022-03-01

**Authors:** Gerson Nakazato, Renata K. T. Kobayashi

**Affiliations:** Center of Biological Sciences, Department of Microbiology, State University of Londrina, Londrina, Brazil

**Keywords:** nanoparticles, strategies, therapy, disease prevention, synthesis, resistance

Nanotechnology has been considered an interesting tool in the chemical, physical, and biological fields to solve many daily problems. With the right strategie, the properties of nanoparticles allow the utilization of this technology to reap the benefits in science and in the industrial process.

Several studies have reported on the use of nanoparticles in Microbiology (diagnosis, vaccine, and antimicrobials). In this Research Topic, different nanoparticles and their applications were focused on antimicrobial activity against bacteria and fungi, including the multi-drug resistant. This Research Topic is a continuation of “*Nanotechnology for Antimicrobials*” with more up-to-date subjects and new descriptions about antimicrobials and nanoparticles-associated.

The global strategies to fight against multidrug-resistant (MDR) pathogens became complex with the COVID-19 pandemic. There has been an increase in hospitalization due to COVID-19, with mechanical ventilation and a prolonged period of hospitalization, representing a high risk for acquiring antimicrobial resistance (AMR). Many countries had troubles with secondary infections in hospitals including multidrug-resistant bacteria and fungi. In addition, there was an increase in the use of antimicrobials in hospitals and in the community. Thus, the impact of COVID-19 on antimicrobial resistance is not yet known but is considered one of the principal threats to global public health according to the World Health Organization (WHO). The need for new antimicrobial agents is urgent, the strategy of developing nanoparticles with antimicrobial action being one of the most promising.

Through this scenario, eight articles of this Research Topic focused the antimicrobial activities and nanoparticles with different features (e.g., nanoparticles kind, synthesis, applications, pathogens, products, and strategies). Despite many studies about nanoparticles as antimicrobials reported in the literature, new data were described in these articles.

Kedziora et al. compared silver ions with silver nanoformulations (nanosilver embedded in TiO_2_ and aqueous dispersion nanosilver) against *E. coli* and mutant derivatives in outer membrane proteins (OmpA, OmpC, OmpF, OmpR, and CusS). The authors found that outer membrane proteins (OMP), not one in particular, but several, are involved in the transport of Ag^+^ and nanoparticles embedded in TiO_2_, but not in aqueous nanosilver dispersion. They also found that silver-ion-resistant strains (*E. coli* J53 and *E. coli* BW25113ompRG596AcusSG1130A) remained sensitive to the tested silver nanoformulations (SNF), such as aqueous nanosilver. Demonstrating differences in the mode of action of Ag+ and silver nanoparticles. Furthermore, based on the results of computational methods, the authors proposed a novel theory for the interaction of silver with outer membrane proteins, where Ag^0^ interacts weakly with amino acids from the inner layers of outer membrane proteins (OmpF and OmpC), allowing Ag^0^ to slide into the cell more efficiently, with a lower energy barrier compared to Ag^+^. Khalil, El-Maghraby et al. and Khalil, El-Raheem et al. showed the importance of silver nanoparticles (biological and chemical syntheses) as antimicrobials through two articles in this Research Topic. The silver nanoparticles synthetized chemically were combined with neomycin and they resulted in synergic antibacterial effect against *Pseudomonas aeruginosa* multidrug-resistant from burn wound infection including the development of drug formulations (gel and spray) for experimental animals. Another study focused on the Biosynthesis of silver nanoparticles by marine actinobacterium *Nocardiopsis dassonvillei*. These biogenic silver nanoparticles showed antimicrobial effect against Gram-positive/Gram-negative bacteria (including multidrug-resistant such as ESBL-producing *E. coli*) and fungi (yeast and filamentous). Interestingly, the authors explored other therapeutic potentials such as antioxidant, insecticidal, and anticancer.

Kaur et al. also approached the battle against multidrug-resistant bacteria in their review on using photothermally active nanomaterials (PANs). PANs are one of the forms of photothermal therapy and include nanomaterials such as gold, carbon, and iron oxide (FeO) that exhibit high absorption in the near-infrared (NIR) region of the electromagnetic spectrum and act by absorbing NIR light and converting it into heat, increasing the temperature around the irradiated site. This review approached the inactivation of multidrug-resistant bacterial using nanoparticles under broad-spectrum irradiation, such as ultraviolet, visible, and infrared light. PANs are alternatives to nanomaterials with metallic bases and metallic oxides, once they present less toxicity to the host and efficient antibacterial action and biocompatibility. PANs target bacterial cell membranes that due to photothermal activity lead to disrupting the microbial membrane and ROS production, showing effective bactericidal activity, they also act on efflux pumps, in addition to inhibiting biofilms and Quorum Sensing. PANs can also be used as drug carriers, acting synergistically when in combination with antibiotics, improving antibacterial activity, and reducing toxic effects. Thus, the authors hold that PANs are of great importance as they are important options for broad-spectrum antibacterial therapies that can combat threatening AMR and contain superbugs.

Zharkova et al. investigated silver nanoparticles (AgNPs) conjugated to antimicrobial peptides or proteins (AMPs/APs) such as protegrin-1, indolicidin, protamine, histones, and lysozyme for their antibacterial properties, including multidrug-resistant strains and their toxic effects against normal and tumor eukaryotic cells. Many conjugates demonstrated significant MIC values reduction when compared to unconjugated nanoparticles. No conjugates caused a drop-in antibacterial activity. Low hemolytic activity was found for all AgNP-AMP/AP conjugates, demonstrating that conjugation is a promising strategy not only to increase their antimicrobial potential but also to effectively reduce the toxicity of membranolytic AMPs.

In addition to bacterial resistance, microbial biofilms are a concern in infectious processes and many studies have approached the prevention or elimination of these biofilms. The antibiofilm effect of iron oxide (Fe_2_O_3_) nanoparticles (IONPs) combined with antibiotics (teicoplanin or vancomycin) against *S. aureus* biofilm was found by Berini et al. Moreover, these authors used an external magnetic field to reduce the intrinsic cytotoxicity toward a human cell line.

Vundela et al. photosynthesized selenium nanoparticles (SeNPs) from Carica papaya fruit and explored their multi-biofunctional applications (e.g., antioxidant, antimicrobial, antimycotoxin, and anticancer activities). These SeNPs exhibited high effect antifungal against mycotoxigenic *Aspergillus ochraceous* and *Penicillium verrucosum*. The biocompatibility of these SeNPs was demonstrated in cell culture (Vero) and *Danio rerio* embryos indicating another benefit for biological purposes.

Ghosh et al. approached nanoformulations associated with crude plant extracts (tulsi, turmeric, oregano, and eucalyptus) and important phytochemicals (eugenol, curcumin, anthraquinone, carvacrol, and eucalyptus oil) with antibacterial potency (Nano-antibacterials). Some strategies were reported in this review such as Nanonization with nanonized (capped and stabilized) products with better therapeutic potential. Throughout, the authors considered the biocampatibility of the nanodrugs in these formulations. In this way, green products with medicinal plant extracts were focused on in this article.

The matter of these articles demonstrates the advantages of Nanotechnology for infection control in different fields, even as model studies for antimicrobial activity. The nanoparticles biosynthesized (“green nanoparticles”) and their biocompatible approach in this Research Topic shows a concern for safety in their uses. Together with products and materials, the synthesis of nanoparticles i.e., biological or green processes was performed in some studies of this Research Topic.

Nanotechnology and Antimicrobials perspectives suggest that infection control is improving over time due to the concern of microbial resistance (superbugs) and the development of new drugs. The Research Topic shows an alternative for conventional antimicrobial therapies using new technologies such as Nanotechnology.

## Author Contributions

All authors listed have made a substantial, direct, and intellectual contribution to the work and approved it for publication.

## Funding

This work was supported in part by the National Council for Scientific and Technological Development—CNPq (process 313305/2019-6) to RK and (process 315435/2018-6) to GN.

## Conflict of Interest

The authors declare that the research was conducted in the absence of any commercial or financial relationships that could be construed as a potential conflict of interest.

## Publisher's Note

All claims expressed in this article are solely those of the authors and do not necessarily represent those of their affiliated organizations, or those of the publisher, the editors and the reviewers. Any product that may be evaluated in this article, or claim that may be made by its manufacturer, is not guaranteed or endorsed by the publisher.

